# A Rare Cause of Walking Disorder in Childhood

**DOI:** 10.5334/jbsr.2813

**Published:** 2022-05-25

**Authors:** Thomas Burguet, Pierre Reginster

**Affiliations:** 1 CHC Mont Legia liège, BE; 2 CHC Mont Legia, BE

**Keywords:** guillain barre syndrome, walk disorder, magnetic resonance imaging

## Abstract

**Teaching point:** Guillain-Barre Syndrome is a rare cause of a walking disorder during childhood. MRI is a key role for diagnosis.

## Case

A 16 months old child was admitted in the emergency department because of a walking disorder evolving since four weeks. The child was in good condition and with no particular medical history. At clinical examination, the child was unable to turn around or to move from a seated to a standing position. He was able to stay in a standing position only with help. The tone of legs was Flabby and there was no spontaneous mobility. Osteo-tendinous reflexes were abolished. There was no pain and no movement limitation.

Blood and cerebrospinal liquid analysis were normal. EMG revealed important decrease of motor conduction velocities.

**Figure 1:**
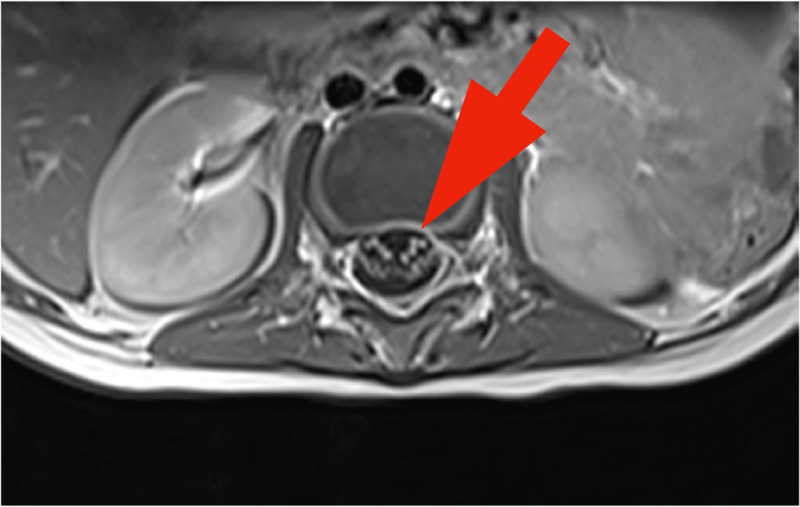


**Figure 2:**
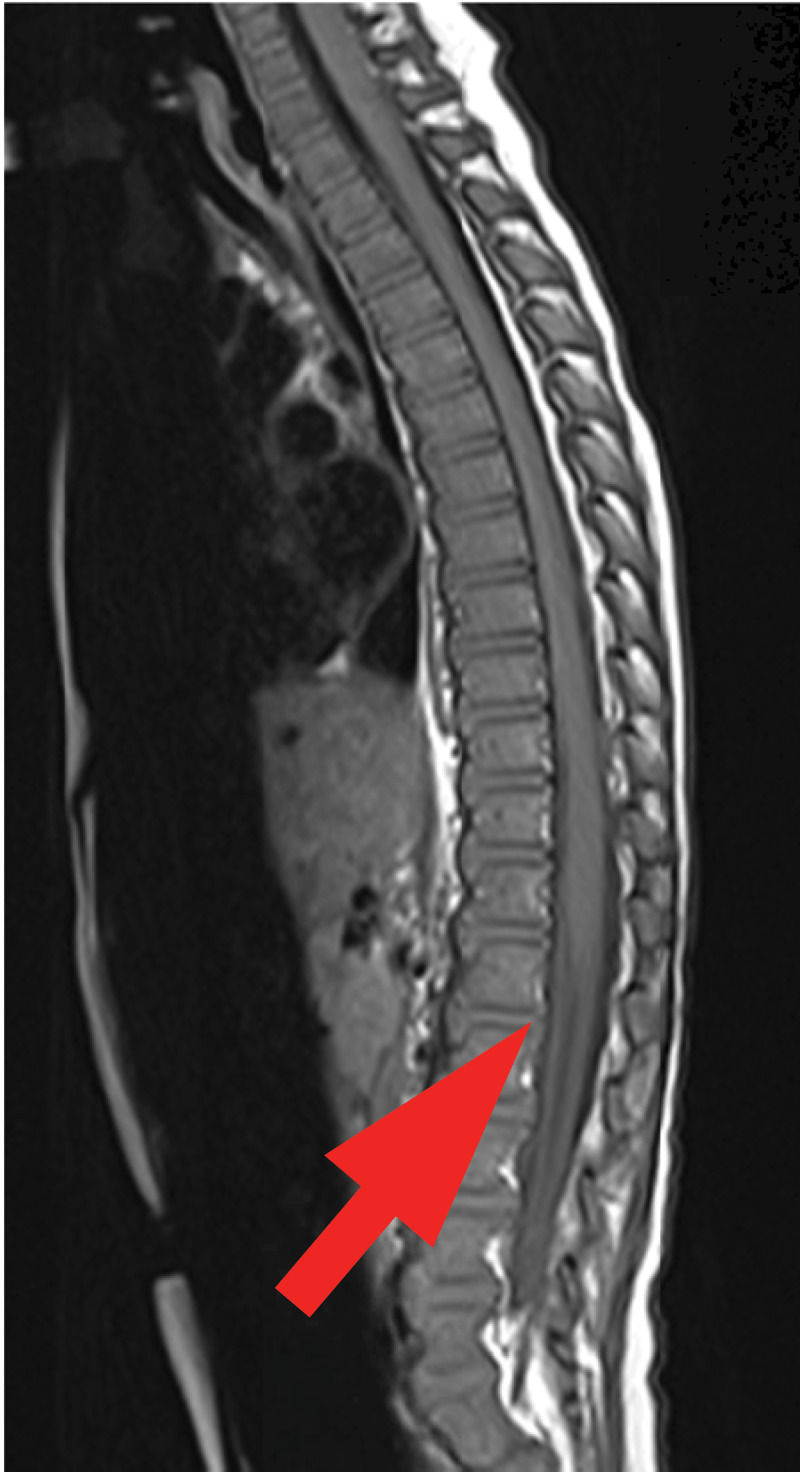


**Figure 3:**
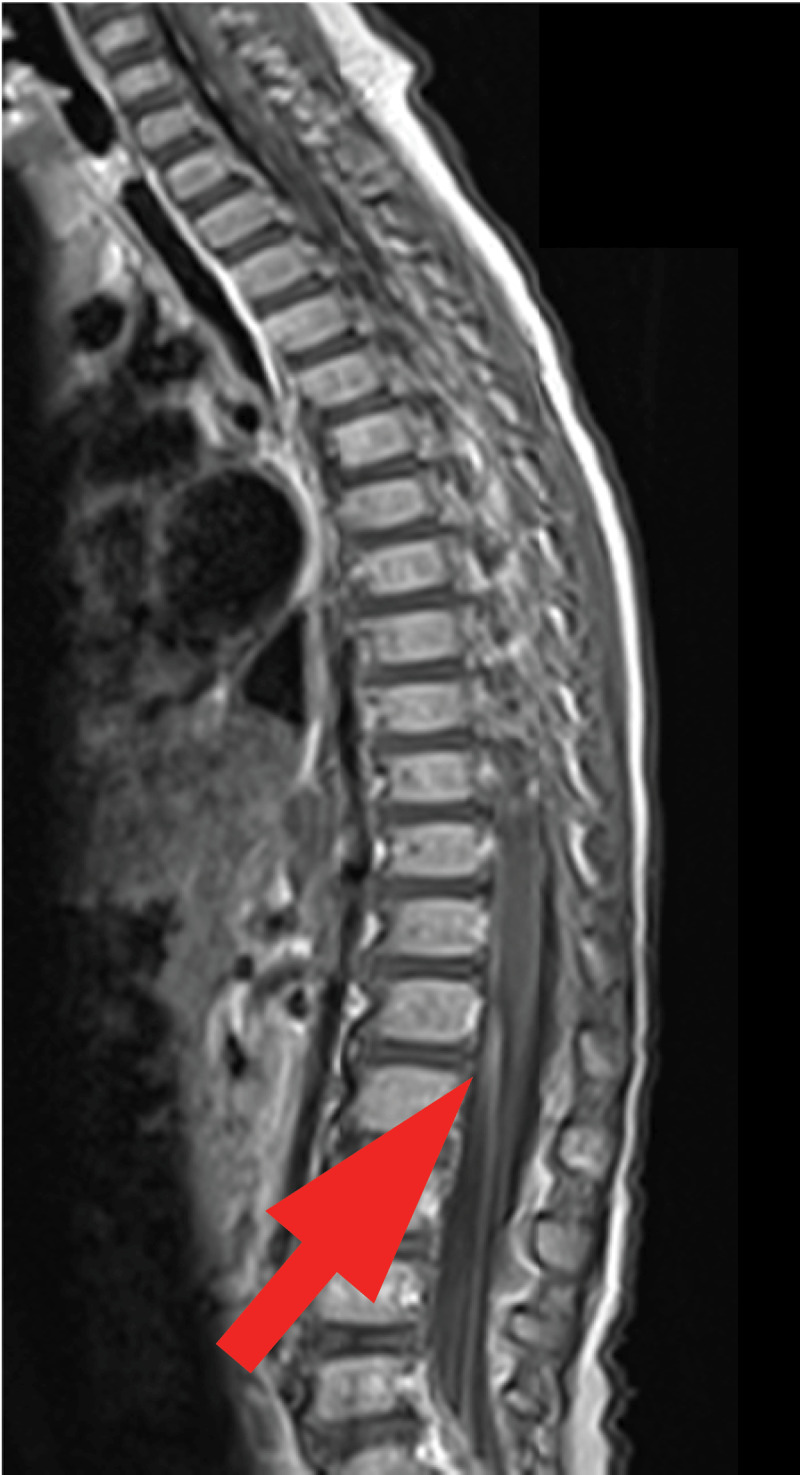


Hip and knee US showed no joint effusion. Magnetic Resonance Imaging of the brain and of the spine showed normal brain and spinal cord. Lumbosacral roots were regularly thickened and strongly enhanced on the post-contrast fat satured T1-weighted sequences (arrow on axial [Figure 1] and sagittal scans [Figures 2, 3]). These characteristics predominated on the anterior roots. The diagnosis of Guillain Barre Syndrome (GBS) was established.

The child was treated by intravenous Gamma globulin administration and physiotherapy.

He recovered well with a normal musculoskeletal behavior after three months.

Further investigation was not conclusive for any etiological factor.

## Comment

GBS is an autoimmune acute inflammatory demyelination of peripheral nerves, nerve roots, and cranial nerves. GBS affects the myelin sheath and reduces the nervous transmission. The incidence in children <18 years is 0,5–1,5:100,000.

There is an attributable ‘trigger’ event in 70% of cases, usually recent viral illness. Vaccination or drug administration can also occur and the presence of Campilobacter jejuni is also often associated.

There is no gender preference and it affects all ages, races and socioeconomics status.

The clinical pattern begins with a distal paraesthesis rapidly followed by ascending paralysis. It is typically bilateral and symmetrical and can ascent up to brainstem to involve cranial nerves. Respiratory paralysis can occur in severe cases. GBS may also have sensory and autonomic disturbances.

Patients usually improve in two to six months. Nevertheless, 30–50% of patients have persistent symptoms at one year, and 5–10% may have permanent deficit. Mortality is reported in up to 8%.

In addition to EMG and analysis of the cerebfrospinal fluid (usually revealing hyperproteinorachia), MRI has a key role in the diagnosis. Nerve roots (cranial and/or spinal) appear slightly thickened and a smooth diffuse (not nodular) enhancement of the nerve roots is observed, mainly on the anterior roots.

Treatment is based on plasma exchange or administration of intravenous gamma globulins. Intensive care management is necessary in severe cases.

